# Abdominal wall endometrioma: An insidious cause of delayed diagnosis

**DOI:** 10.1002/ccr3.4836

**Published:** 2021-09-15

**Authors:** Sofoklis Stavros, Ekaterini Domali, Ioannis K. Papapanagiotou, Peter Drakakis, Alexandros Rodolakis

**Affiliations:** ^1^ Department of Obstetrics and Gynecology Alexandra Hospital National and Kapodistrian University of Athens Athens Greece

**Keywords:** abdominal wall, cesarean section, endometriosis, translabial ultrasonography

## Abstract

An extremely rare extrapelvic position of endometriosis with a precise incidence of 0.07%–0.47%, leading usually to delayed and false diagnosis. Differential diagnosis should include that rare condition while ultrasonography remains a pivotal tool to unravel that enigma, especially in women with no specific symptoms and surgeries in the past.

## 1 INTRODUCTION

Abdominal wall endometrioma is an extremely rare entity with a precise incidence of 0.07%–0.47% remaining an insidious cause of usually delayed diagnosis. Differential diagnosis should include that rare condition and ultrasonography remains a pivotal tool to unravel that enigma as well.

**FIGURE 1:**
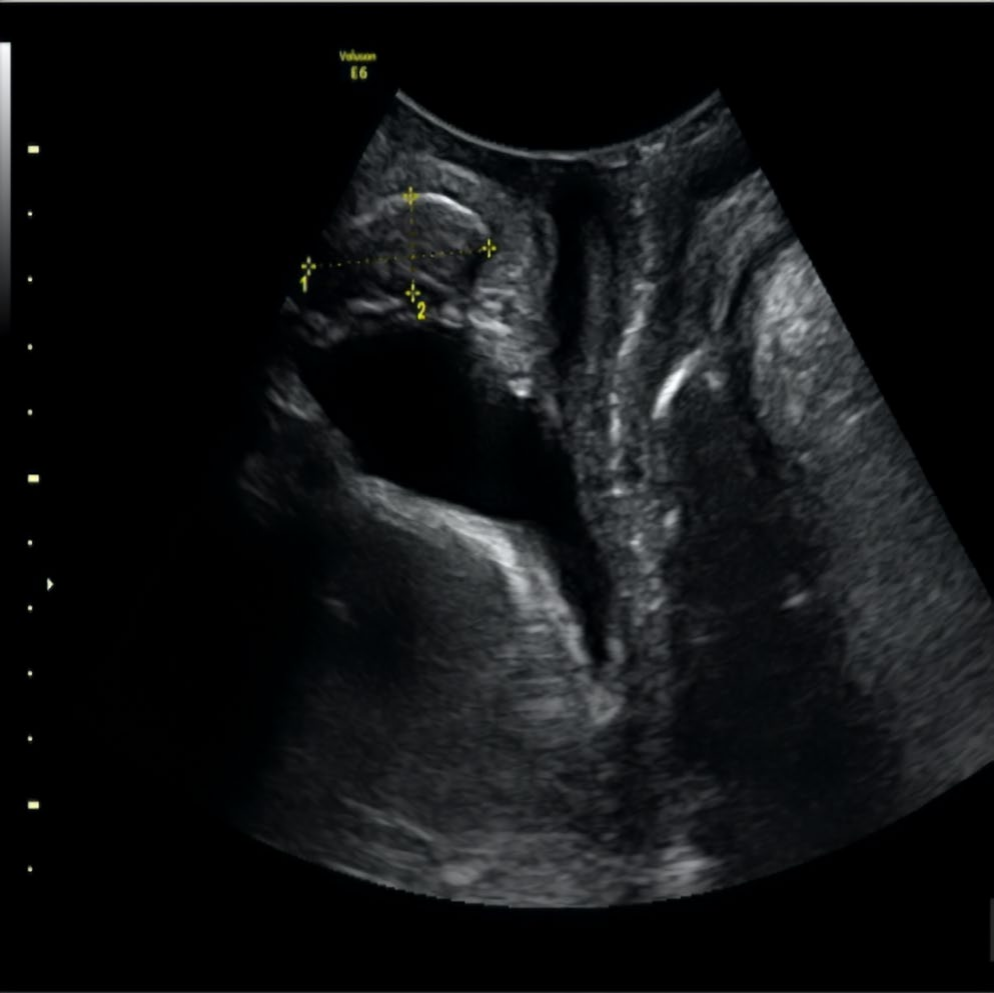
Translabial ultrasonic image; a lesion of 36 × 15 mm is being present in the median tissue between the abdominal wall and the subcutaneous tissue

**FIGURE 2:**
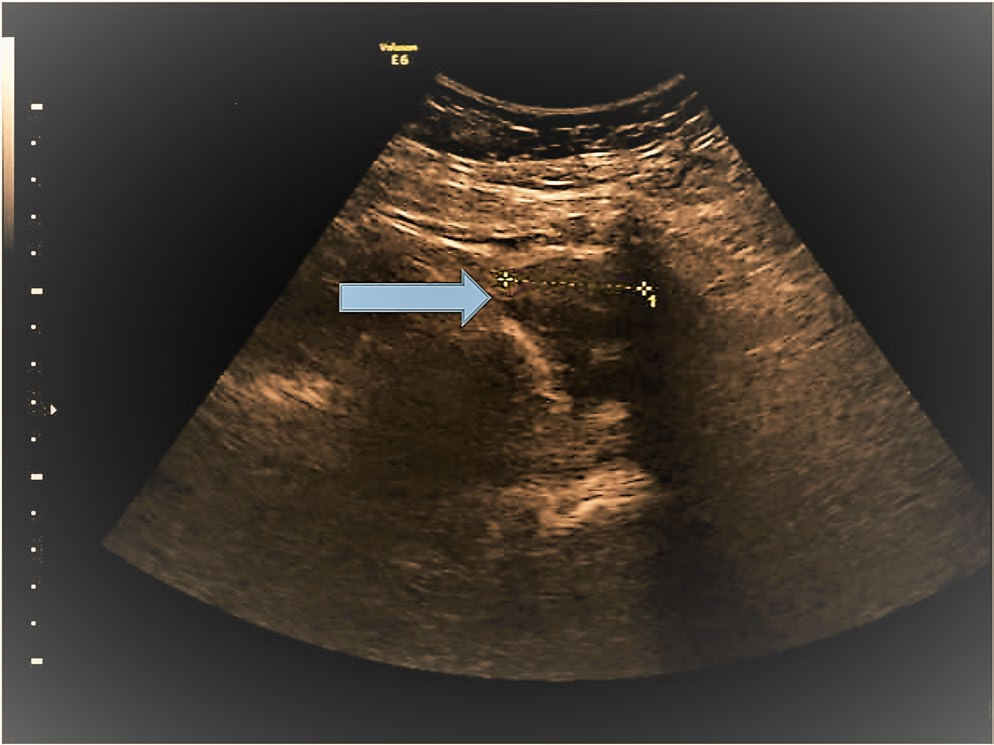
Hypogenic lesion underneath the scar of the abdominal wall with clear margins and benign characteristics via the transabdominal ultrasonography

A 34 years old woman was presented 4 years ago to our facility complaining for pain due to a palpable nodule in the abdominal wall of previous cesarean sections. No other symptom was reported. She had a personal history of two cesarean sections (2007 and 2012), and a laparoscopic surgery for ruptured ovarian cyst in 2013. Clinical examination revealed a palpable lesion in the left abdominal side and precisely into the abdominal wall. Vaginal examination and laboratory examinations were all normal. Transabdominal and translabial ultrasonography were performed (Figures 1 and 2). An intra‐abdominal hypogenic lesion of 36 × 15 mm was unraveled via the translabial ultrasound in the median tissue between the abdominal wall and the subcutaneous tissue. Hence, a surgical removal under general anesthesia was performed. Histological diagnosis verified that insidious lesion‐endometrioma. Cesarean section and hysterotomy are the most common operations associated with abdominal wall endometriosis. Pfannenstiel scar remains the most common site for extra pelvic endometriosis with a precise incidence of 0.07%–0.47%.^1^ No specific findings and symptoms lead usually to delayed diagnosis. Granuloma, lipoma, abscesses, sebaceous cysts, ventral hernias, or metastasis should be included in the differential diagnosis of those lesions.^2^

## CONFLICTS OF INTEREST

The authors declare that they have no competing interest.

## AUTHOR CONTRIBUTIONS

SS made substantial contribution to acquisition of data. SS, ED, and IP made substantial contribution to conception, analyzing, and drafting the manuscript. SS contributed in analyzing data and revising the manuscript. IP contributed in acquisition of the data. ED: contributed in analyzing data. PD revised the manuscript. PD and AR agreed to be accountable for all aspects of the work. PD and AR gave final approval of the version to be published.

## ETHICAL APPROVAL

Patient consent has been collected. The Ethics Committee of the Hospital has approved this Clinical Image.

## CONSENT

The authors have confirmed during submission that patient consent has been signed and collected in accordance with the journal's patient consent policy.

## Data Availability

Data openly available in a public repository that issues datasets with DOIs.
